# Evaluating the photocatalytic performance of MOF coated on glass for degradation of gaseous styrene under visible light

**DOI:** 10.1038/s41598-023-51098-w

**Published:** 2024-01-11

**Authors:** Zohre Kazemi, Ahmad Jonidi Jafari, Majid Kermani, Roshanak Rezaei Kalantary

**Affiliations:** 1 Research Center of Environmental Health Technology, Iran University of Medical Sciences, Tehran, Iran; 2 Department of Environmental Health Engineering, School of Public Health, Iran University of Medical Sciences, Tehran, Iran

**Keywords:** Environmental sciences, Chemistry

## Abstract

Styrene is a volatile organic compound with various applications, especially in the plastics and paint industries. Exposure to it leads to symptoms such as weakness, suppression of the central nervous system, and nausea, and prolonged exposure to it increases the risk of cancer. Its removal from the air is a topic that researchers have considered. Various methods such as absorption, membrane separation, thermal and catalytic oxidation, biofiltration have been used to remove these compounds. The disadvantages of these compounds include the need for high energy, production of secondary pollutants, large space, providing environmental conditions (temperature and humidity) and long time. The photocatalyst process is considered as an advanced process due to the production of low and safe secondary pollutants. MOFs are nanoparticles with unique photocatalytic properties that convert organic pollutants into water and carbon dioxide under light irradiation and in environmental conditions, which prevent the production of secondary pollutants. The present study aimed to investigate the efficiency of MIL100 (Fe) nanoparticles coated on glass in removing styrene vapor from the air. Surface morphology, crystal structure, pore size, functional groups, and chemical composition of the catalyst were analyzed by SEM, XRD, BET, FTIR, and EDX analysis. The effect of parameters such as initial pollutant concentration, temperature, time, relative humidity, and nanoparticle concentration was evaluated as effective parameters in the removal process. Based on the results, MIL100 (Fe) 0.6 g/l with an 89% removal rate had the best performance for styrene removal. Due to its optimal removal efficiency, it can be used to degrade other air pollutants.

## Introduction

Due to the rapid growth of the economy and industrialization, air pollution is one of the most important threats to human societies, including endangering health, death and mortality, hospitalization due to respiratory and cardiovascular diseases, cancer, and climate change^1^. One of the main public health goals in developing countries is to reduce air pollutants to reduce the mortality caused by them^2^. Volatile organic compounds (VOCs) are dangerous pollutants released into the air and are released from various sources, such as the oil industry, refineries, vehicles, household products, paints, plastics, and silicon manufacturing processes plants. Exposure to small amounts of these compounds (less than 100 ppmv) for a long time causes some problems such as cancer, liver and kidney failure, as well as respiratory diseases^3–6^. The presence of these compounds in the smoke leads to eye irritation and respiratory problems^7^. Such compounds can react with nitrogen oxides in sunlight to form ozone, peroxyacetyl nitrate, and polycyclic aromatic hydrocarbons (PAHs). These pollutants are involved in the formation of some particles such as PM_2.5_ and photochemical smog^8,9^, which leads to reduced air quality^10^. Styrene is a volatile organic compound in the production of fiberglass, laminate, insulation, auto parts, car tires, foam, resin, polyester, and plastic bottles. It is present in small amounts in food, tobacco smoke, and car exhaust fumes. The most important uses of styrene are in the rubber and plastic industries^11–14^. Industrial activities and exhaust fumes of motor vehicles are the main factors of their emission into the atmosphere^14^ and it is absorbed more by inhalation through the skin^14,15^. The highest exposure rate is found in occupations, during a study, its level was observed in the blood of workers 25 times more than in other people^14^. A study reported that occupational environments, especially industries producing reinforced plastic products, had the highest exposure to this pollutant^13^. The ACGIH standard states that the occupational exposure limit for styrene is 20 ppm for 8 h of daily work and 40 h per week^16^. According to the Environmental Protection Agency, exposure to styrene can lead to some problems such as suppression of the central nervous system, loss of concentration, weakness, fatigue, and nausea. Prolonged exposure can damage the reproductive system. In addition to the above, eye and nose irritation, headache, vertigo, dizziness, ataxia, dermatitis, and liver damage have been listed as complications of exposure to it ^7,15^. The International Agency for Research on Cancer has classified it in the category 2B in the sense that it can possibly cause carcinogenesis in humans^17^. A recent American study reported a significant association between styrene and the prevalence of neurological symptoms ^12^. Kanwal and Boylstein also found pollutants such as formaldehyde, acetic acid, and styrene in the air at the automotive assembly plant, leading to asthma, respiratory irritation, and bronchitis ^18^. Mohamadyan et al., in their study on the exposure to styrene and its metabolites in people working in plastic factories, found that the average exposure to styrene was 83.2 ± 32.4 mg m^−3^, and the amount of mandelic acid in urine was 1570.1 ± 720.6 mg g creatinine^−1^^15^. Toxicological studies have shown several neurological effects due to exposure to styrene ^13^. The VOCs are controlled using adsorption, catalyst, thermal oxidation, and photocatalytic oxidation methods ^11^. Efficient removal of these pollutants in the environment has become a hot topic nowadays. Various technologies such as membrane separation, thermal and catalytic oxidation, biofiltration, etc., are employed to treat environmental pollutants. Many of these methods have disadvantages such as low efficiency, production of secondary pollutants, high energy consumption, and provision of suitable conditions. Adsorption and photocatalytic processes have been proposed as cost-effective measures for treating environmental pollutants due to their low cost, low production of secondary pollutants, and safe nature. The photocatalyst process is extensively used due to its application in environmental conditions, the production of hydroxyl radicals and the degradation of toxic organic matter to non-toxic minerals such as water and carbon dioxide, and the decomposition of pollutants without the need for auxiliary chemicals such as ozone^6,11,19–22^. So far, from various absorbers such as; Zeolites, silica, activated carbon, graphene and polymer have been used to remove these pollutants^3,5,6,19,20,23^. The limitations of these adsorbents, we can mention the chemical reaction between the pollutant and the adsorbent, high energy, small pore size and its blocking, instability against moisture and changing its properties at high temperature. Also, after some time of use, absorbents must be regenerated. In the thermal regeneration process, the absorbed pollutant is released into the air and secondary pollution occurs ^3,6,23^. One of the catalysts used is platinum (Pt). The use of this nanoparticle was limited due to the high cost of providing the energy needed to heat it, its price and lack of abundance in nature, and nickel (Ni), copper (Cu) and gold (Au) were used as alternatives ^6^. Some catalysts are also activated at high temperatures and cannot be used at ambient temperature ^24^. The use of nanoparticles such as SnO_2_, WO_3_, CdS, ZnO, TiO_2_, etc., due to the small area, low absorption capacity, slowness Reaction speed, cost and deposition of intermediate products resulting from degradation on the catalyst were limited and reduced its efficiency^11,25–28^. Some of these nanoparticles are unstable when combined with fibers or plastic in the form of a composite, and after some time they decompose spontaneously under sunlight ^21^. The use of some common nanoparticles has been reduced due to limitations such as the activation of the catalyst under ultraviolet light, which includes only 4–5% of the total energy of the sun ^29^. High-efficiency porous nanoparticles have become important issue to improve the environment. Metal–organic frameworks (MOFs) are among these nanoparticles introduced in the mid-1990s as crystalline porous materials with high chemical diversity and high surface area^30,31^. These frames are made of metal cations (nodes) and organic ligands^32^. These structures are used in many cases such as gasoline storage and separation, catalyst, as well as chemical assays due to having large size holes, high specific surface area, the ability to choose organic and inorganic compounds, crystalline structure, the possibility of adjusting the pore size, non-toxicity, low cost, etc., are used as absorbers of gaseous pollutants^1,30,32–37^. These

## Materials and methods

### Synthesis of MIL100(Fe)

**Figure 1:**
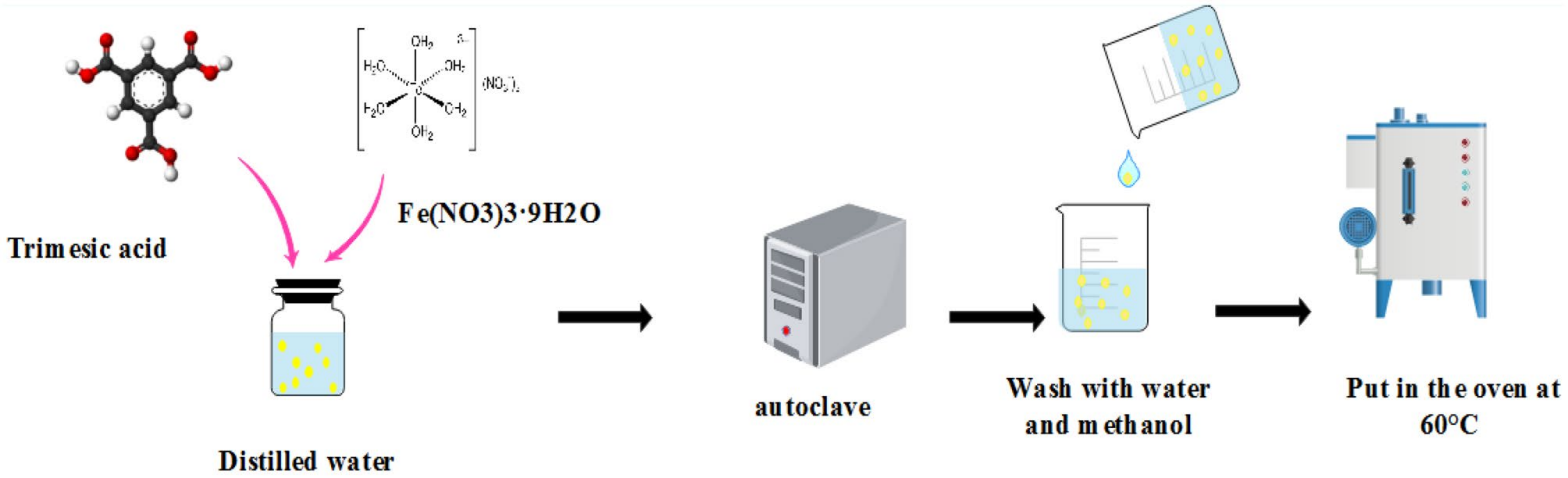
Steps in the synthesis of nanoparticles MIL100(Fe).

In the first stage, 0.484 g of Fe(NO_3_)_3_·9H_2_O (CAS NO: 7782-61-8, 99% Merk) and 0.21 g of C_9_H_6_O_6_ (CAS NO:554-95-0, ˃ 99% Sigma-Aldrich) were poured into some of distilled water. After stirring, the mixture was heated in an autoclave (REYHAN TEB, 56 HZ frequency) at 180 °C for 12 h. The made material was washed several times with water and methanol (CAS NO:67-56-1, 99% Merk) and dried in an oven at 60 °C^40^ (Fig. 1).

### Characterization

After fabrication of the catalyst, the surface and pore volume were investigated by BET analysis (JW-BK132F). In order to check the BET analysis of the synthesized catalyst, first the sample was degassed at 150℃ for 16 h under vacuum. BET method was calculated in the range of relative pressure P_0_ = 0.05–0.20 and pore volume at relative pressure 0.95 The FTIR analysis (Irsolution 8400 S) was also used to determine the available functional groups. In order to analyze, 1 mg of nanoparticles was mixed with KBr material. Then it was pelletized and pressed and analyzed in Nicolet 6700 spectrometer made by THERMO company in America in the range of 400–4000 cm^-1^. Catalyst form was observed by SEM analysis (VEGA 15 kV, Manufactured by TESCAN, Czech Republic). Catalyst constituents was determined by SEM–EDX analysis. The crystal structure of MOF was determined by XRD analysis (Philips PW170) with X-ray irradiation at CuKα (λ = 1.5406 Å), voltage was 35 kV, the emission current was 40 mA and the scanning speed was 0.1 s^−1^ in the range of 5°–80°.

### Photocatalytic experiment

**Figure 2:**
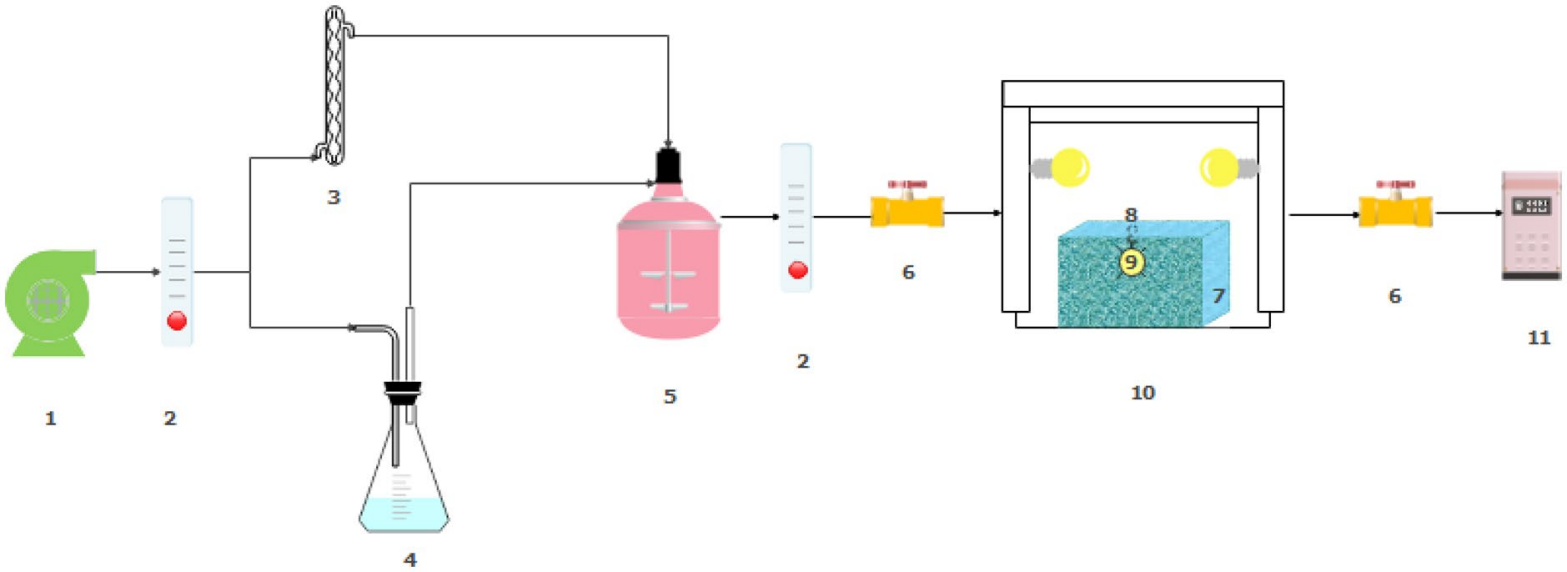
Schematic diagram of continuous flow reactor for photocatalysis. 1. Air pump. 2. Rotameter. 3. Impinger containing pollutant. 4. Water container. 5. Mixing tank. 6. Control valve. 7. Photocatalytic reactor. 8. Quartz jacket. 9. IR lamp. 10. Plexiglas box. 11. Analyzer.

Figure 2 indicates a system used to evaluate the removal efficiency of styrene. The area of glasses used was 12 × 25 cm^2^, 17 × 25 cm^2^, and 22 × 25 cm^2^. Inside the reactor, two fans were used to circulate the volatile organic compound and react it with nanoparticles. The quartz reactor with dimensions of 20 cm × 26 cm × 30 cm was connected to the direct reading device of Phocheck Tiger (model 5000 made in England). The measuring range of volatile organic compounds in this device is in the range of 0.1 to 10,000 ppm. A xenon 55 w (λ = 475 nm) lamp was installed inside the quartz jacket on top of the reactor to perform the photocatalytic process. The reactor was covered with aluminum foil to prevent other light from entering the reactor. Two lamps were installed on both sides of the box to regulate the temperature. Parameters such as changes in nanoparticle and pollutant concentrations, temperature, relative humidity, and time were examined. First, the reactor was purified with a suction pump. The inlet airflow was divided into two parts by a blower pump (Aquarium Air Pump SB848), part of which entered the emitter containing styrene pollutant. The other part was passed through the Erlenmeyer containing water to provide humidity. The vapors from these two parts entered the mixing chamber equipped with a fan. Styrene gas with concentrations of 50 ppm, 100 ppm, and 150 ppm, relative humidity of 15%, 30%, and 45%, and temperatures of 20 °C, 25 °C, and 35 °C was injected into the reactor containing glass coated with MIL100 (Fe) nanoparticles. The reactor was kept in the dark for 15 min (proportional to the flow rate and volume of the reactor) to achieve an adsorption–desorption equilibrium between the catalyst and styrene. Then, the reactor was sampled for photocatalytic degradation at 30 min, 60 min, 90 min, and 120 min under visible light. All experiments were performed twice. In order to ensure the accuracy of the measured results, a sample was injected into a gas chromatography device (Agilent model 7890B, Agilent-MS 597B, MODEL [HI] EI) equipped with an FID detector. The removal efficiency was calculated by using Eq. (1):

1 $${\text{X}} = \frac{{C_{in} - C_{out} }}{{C_{in} }} \times 100$$

(C_in_) = input concentration, (C_out_) = output concentration.

### Ethical approval

The authors of this article have covered all the ethical points, including non-plagiarism, duplicate publishing, data distortion, and data creation in this article. This project has been registered in Iran University of Medical Sciences with the code of ethics of IR.IUMS.REC.1399.254.

## Results and discussion

### Characterization of MIL100(Fe)

**Figure 3:**
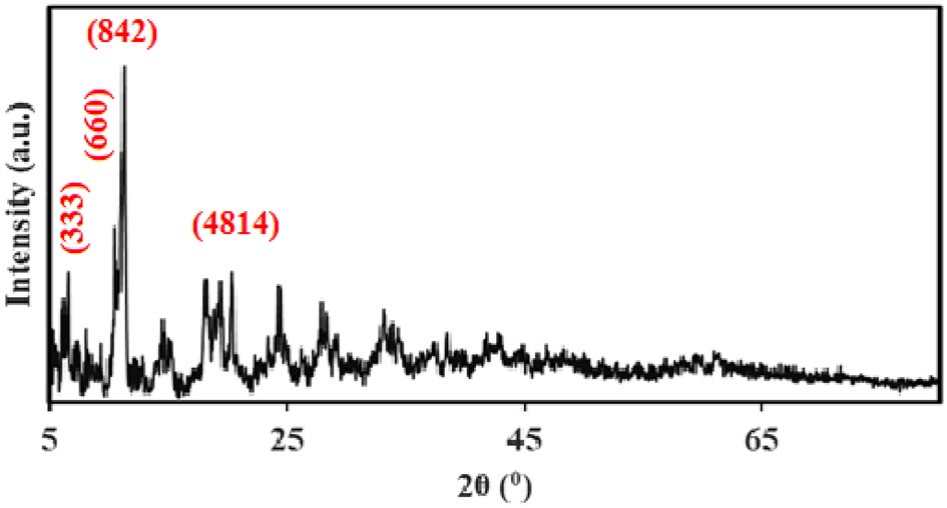
XRD of MIL100 (Fe).

**Figure 4:**
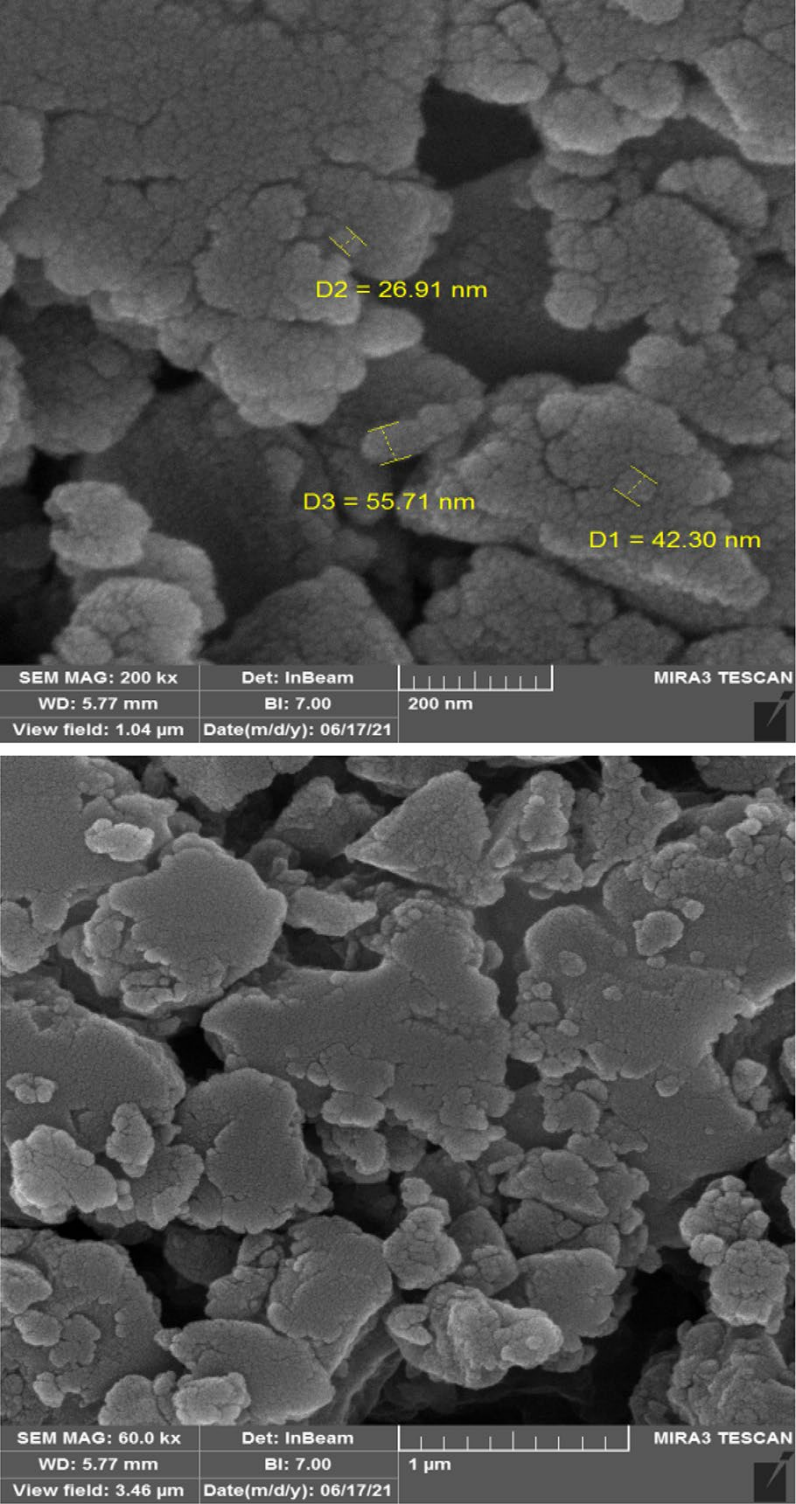
SEM of MIL 100 (Fe).

**Figure 5:**
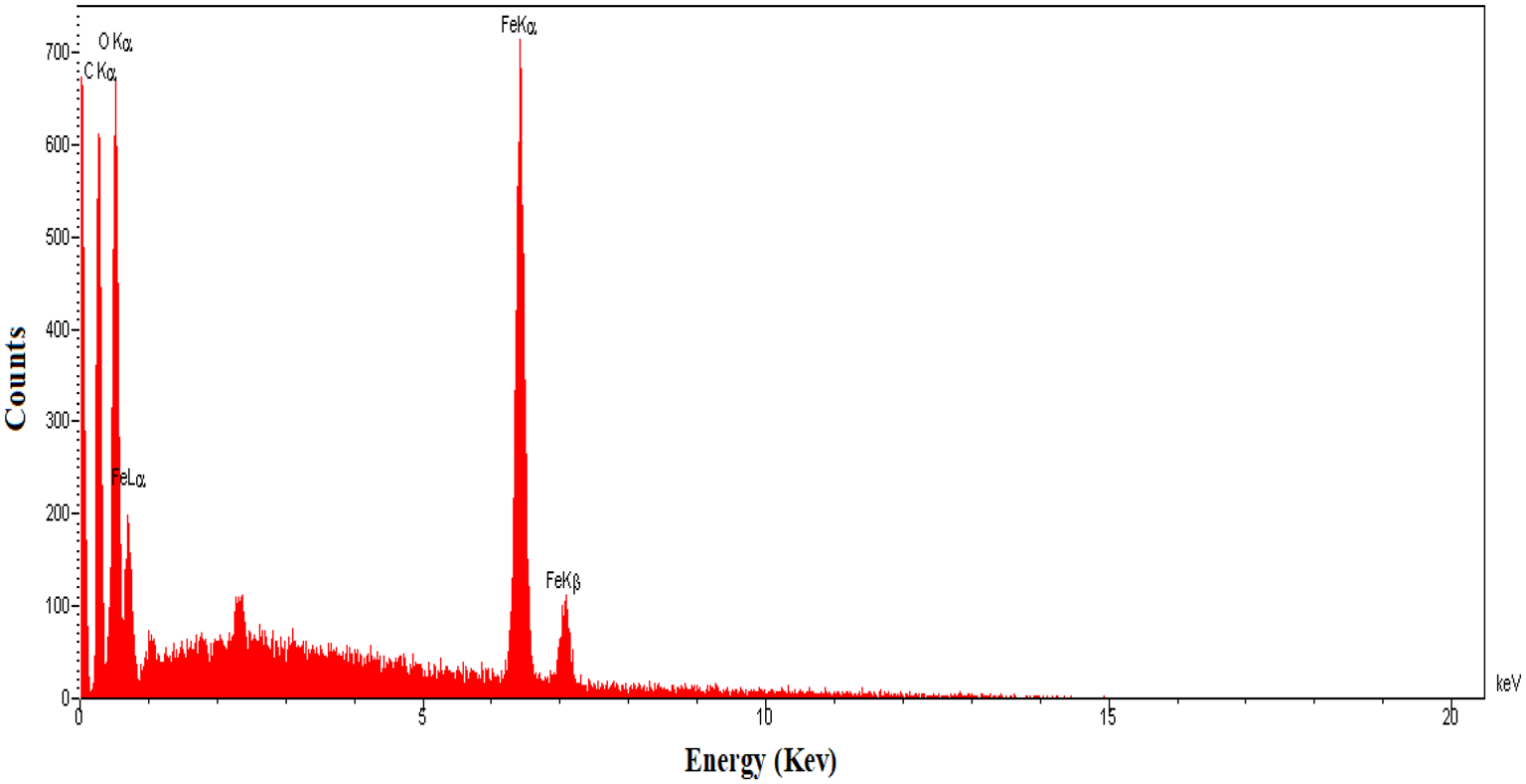
EDX of MIL100 (Fe).

**Figure 6:**
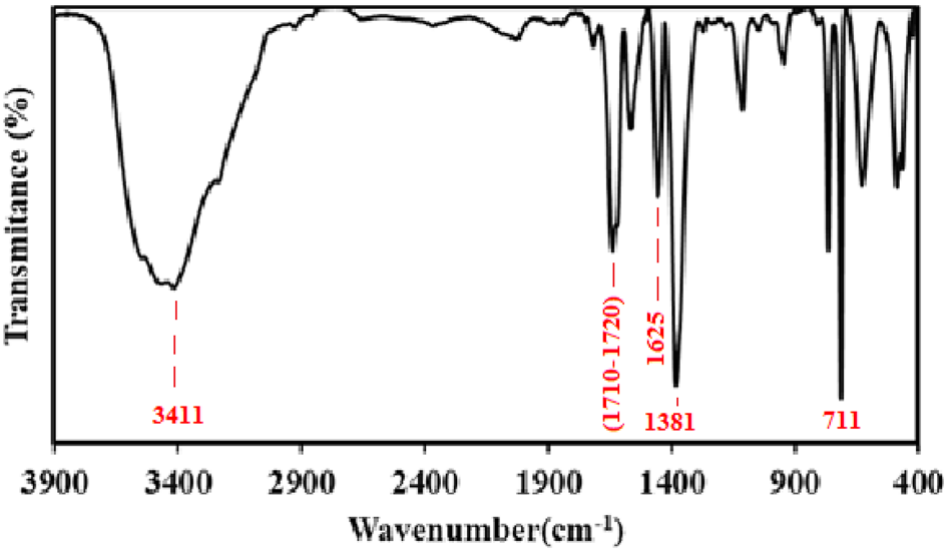
FT-IR of MIL 100 (Fe).

According to the X-ray diffraction in Fig. 3, the 2θ = 6.3°, 10.2°, 11.0°, and 20.0° correspond to the (333), (660), (842), and (4814) planes of MIL100 (Fe), respectively, which is in line with other studies^30,31,35,41–43^. Like other studies, the main peak of approximately 10 degrees, which is one of the main symptom of MIL100 (Fe) nanoparticles, can be seen in the diffraction pattern ^44^. Guesh et al. observed that the main peak in MIL100(Fe) structure is 2θ = 9°–10° ^31^. Chen et al. also stated that 2θ = 10° is the most prominent peak in MIL100(Fe) nanoparticles^28^. In their study, Cui et al. also observed 2θ = 6.3°, 10.2°, 11.0°, 20.0° peaks in the synthesized MIL100(Fe) structure^41^. Also, in another study conducted by Du et al. reported that 2θ = 6.2°, 10.2°, 11.0°, 20.0° peaks were observed in the synthesized MIL100(Fe) catalyst^43^. The SEM image (Fig. 4) indicated the fineness of the particles and their nano-porosity and it was consistent with the reported structures^30,44,45^. In their study, Wang et al. found the size of the synthesized MIL100(Fe) nanoparticles to be 500 nm^30^. This difference can be due to the type of materials used and the method of synthesis. For example, Wang et al. used FeCl_3_6H_2_O in their research to synthesize MIL100(Fe)^30^. As shown in Fig. 5, the EDX spectrum showed that Fe (36.89%), C (34.37%), and O (28.74%) are the composing elements of MIL100 (Fe), as reported in a research piece^30^. Mohammadifard et al. in a study to remove formaldehyde using MIL100(Fe) catalyst, observed in the EDX analysis of the catalyst that Fe, C and O elements are its main components^46^. In the FTIR analysis, the peaks observed in 1381 cm^−1^ and 1625 cm^−1^ regions are related to bands of O=C–O^−^ trimesic acid (TMA) ligands with Fe (III). The peaks observed in the 711 cm^−1^ and 3411 cm^−1^ regions are related to the Fe–O and O–H bonds. The intensity at 1710–1720 cm^−1^ is related to the C=O of the TMA tensile transitions and it is similar to the studies previously reported (Fig. 6). In the FTIR diffraction of MIL100(Fe) catalyst, the observed peaks in the region of 3445 cm^−1^, 1385 cm^−1^ and 1625 cm^−1^ were attributed to O–H, C–O^−^ and C=O groups^35^. They stated that the peak observed in the region of 1710–1720 corresponds to C=O^8,35,47–49^. Abdpour et al. peaks of 3445, 1629, 1450, 1378 and 711 cm^−1^ in the synthesized MIL100(Fe) nanoparticle are related to O–H, C=O, O–H, C–O and Fe–O bonds^48^. The BET analysis was measured under nitrogen gas. The size of specific surface area, average hole diameter, and hole volume are given in Table 1. The high volume and the particle size of about 26–55 nm of the pores can increase the degradation efficiency. In the study conducted by Liu et al. to remove color with the help of TiO_2_-MIL100(Fe) nanocomposite, the specific surface area of MIL100(Fe) catalyst was 1669.5 m^2^/g^50^. In another study, Guesh et al. reported the specific surface area of MIL100(Fe) catalyst as 2028 m^2^/g^31^. In their research, Huang et al. expressed the specific surface area of the synthesized catalyst as 1650 m^2^/g^35^. Also, in another study, Shi et al. reported the specific surface area of MIL100(Fe) nanoparticles as 1754 m^2^/g^47^. The difference in the specific surface area observed in the studies may be due to the type of materials used for the synthesis of the catalyst preparation method. Researchers have stated that the high specific surface allows for the absorption of more pollutants and hydroxyl radicals resulting from the photocatalytic reaction. Suligoj et al. also observed that the high specific surface area of the catalyst led to the acceleration of the degradation of volatile organic compounds in the air ^51^. The results confirm the synthesized MIL100 (Fe) nanoparticles.

**Table 1:** BET analysis of MIL 100 (Fe).

MOF	Surface area (m^2^/g)	Pore volume (cm^3^/g)	Pore size (nm)
MIL100(Fe)	1814	1.3725	3.0255

### Photocatalytic process analysis of MIL100(Fe)

#### Effect of concentrations of styrene

**Figure 7:**
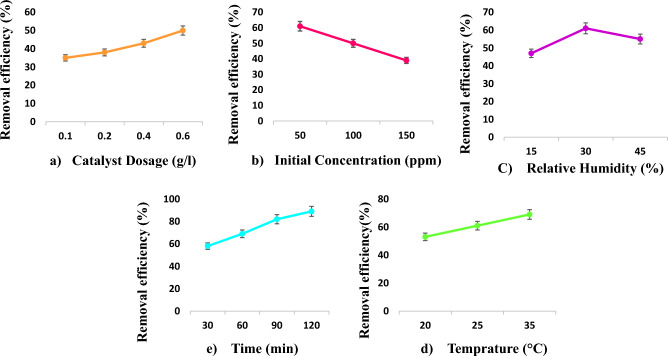
Effect of (**a**) catalyst dosage, (**b**) styrene concentration, (c) relative humidity, d) temperature and e) time on the degradation of styrene.

One of the important parameters in achieving a significant amount of pollutant removal is its initial concentration. In this study, concentrations of 50 ppm, 100 ppm, and 150 ppm were selected based on the exposure to different pollutants in different articles and standards. With increasing the pollutant concentration from 50 to 150 ppm, the removal efficiency decreased from 61 to 39%, respectively, because in the process of photocatalytic degradation, the pollutant is first adsorbed on the active nanoparticle sites and then decomposed. Therefore, as the pollutant concentration increases, fewer photons can penetrate the active parts of the nanoparticles, and the removal rate decreases. In addition, during the decomposition of the pollutant, other by-products are produced that compete with the pollutant molecules for accessing active sites. In line with the results of this study, it was also observed that increasing the concentration of tetracycline pollutants reduced the removal efficiency from 88.2% (C_0_ = 10 mg/L) to 66% (C_0_ = 50 mg/L) ^30^. In addition, Mahmoodi et al. found that increasing the concentration of pollutants such as dyes reduces the degradation due to the production of intermediate products ^45^. Rangkooy et al. also stated that increasing the styrene concentration accelerates the photocatalytic reaction rate but reduces its removal efficiency; pollutant molecules close active sites at the catalyst surface and reduce the production of hydroxyl radicals. In addition, increasing the concentration of pollutants prevents photons from hitting the catalyst's surface, reducing the degradation efficiency ^11^. In another study by Nakhaei Pour et al. with the aim of removing styrene by ZnO nanoparticles coated on an activated carbon adsorbent, it was found that with the increase in styrene concentration, the removal efficiency decreased ^52^. Furthermore, another study aimed to remove styrene by ZnO nanoparticles stabilized on activated carbon zeolites, Y and ZSM-5 found that the catalytic degradation efficiency decreased by increasing the pollutant concentration from 20 to 100 ppm and 300 ppm. It was stated in this investigation that this decrease is due to an increase in the number of pollutants compared to the active sites, blockage of these sites, and a decrease in the hydroxyl radicals production^27^ (Fig. 7). Sun Kim et al. in a study they conducted to remove styrene by biological filters impregnated with C–TiO_2_ composite, observed that by reducing the amount of input styrene by 630, 420 and 105 mg/m^3^, the removal efficiency was 20.6%, increased to 29.8% and 40% ^53^. In a research conducted by Nakhaei Pour et al. it was observed that the removal efficiency decreased from 86 to 34% by increasing the input styrene concentration from 20 to 300 mg/l^26^. Also, Rangkooy et al., in a study they conducted to remove formaldehyde using ZnO-99BC and ZnO-95BC catalysts, observed that the removal efficiency decreased with increasing pollutant concentration^26^. Also, in a study to remove styrene by electron beam radiation, Suk Son et al. observed that at a concentration of 50 ppmv, the removal efficiency was more than 98%, and by increasing the pollutant concentration to 200 ppmv, the removal efficiency was 63%^54^. Various researches have reported that with the increase in pollutant concentration, the number of pollutant molecules increases compared to the active sites of the catalyst, and the molecules are absorbed by the adsorbent at a faster rate and in a shorter time, causing saturation of the adsorbent and reducing degradation^55^. In general, the results of this study indicated that MIL100 (Fe) has an excellent performance in removing styrene.

#### The effect of nanoparticle concentration

As indicated in Fig. 7, with increasing the concentration of nanoparticles, the pollutant removal efficiency increased so that 35%, 38%, 43%, and 50% styrene were removed at concentrations of 0.1 g/l, 0.2 g/l, 0.4 g/l, and 0.6 g/l of MIL100 (Fe), respectively. Initially, when the concentration increased from 0.1 g/l to 0.2 g/l, no significant change in the removal rate was observed. However, the removal efficiency increased significantly by increasing the catalyst dosage to 0.4 g/l and 0.6 g/l. According to the results, a concentration of 0.6 g/l of MIL100 (Fe) removes the most styrene with an initial concentration (50 ppm), which is important for its application in industry. In line with the results of the present study, Wang et al. demonstrated that by increasing the dosages of MIL101 (Fe) nanoparticles from 0.1 g/l to 0.5 g/l, the removal efficiency of tetracycline increased from 32.7% to 55% after 60 min^30^. Moreover, in another study by Mahmoodi et al. with the objective of analyzing dyes using MILs-100, it was found that ultraviolet light could only decompose less than 20% of dyes. However, with the addition of catalysts and light radiation, the degradation rate increased significantly due to the increased production of hydroxyl radicals in the aqueous medium. They also stated that the dye removal efficiency increased with increasing the catalyst dosage due to the increase in the available active surface ^45^.

According to Rangkooy et al.'s study on photocatalytic styrene removal by ZnO nanoparticles with weight percentages of 3%, 5%, and 10%, and stabilized on zeolites modified with hydrochloric acid and diphenyl dichlorosilane, the removal efficiencies of 3% UV/MZe-ZnO, 5% UV/MZe-ZnO and 10% UV/MZe-ZnO were 36.5%, 40%, and 26%, respectively. It shows that initially, with increasing catalyst dose, the number of active sites and subsequently the number of active radicals at the catalyst surface and removal efficiency increased; however, due to the filling (closing) of the holes in higher concentrations, a decrease in the rate of degradation was observed with an increase in the weight percentage of nanoparticles. This effect was due to a decrease in the contact surface of styrene and catalyst and consequently a decrease in styrene adsorption. Another reason is that with increasing the concentration of nanoparticles, the amount of light penetration to active surfaces (an important factor in photocatalytic degradation) is reduced, and the amount of degradation is also decreased ^11^. Tokumura et al. conducted a study on removing acetaldehyde with 1000 ppb of input pollutant by wet scrubber and photo-Fenton reaction and claimed that by increasing the iron concentration from 0 to 50 mg/l, the amount of exhaust gas was significantly reduced. They also stated that photo-Fenton reactions and gas degradation efficiency increased with increasing iron concentration ^56^. In another study, Jeong et al. reported that the acetaldehyde removal efficiency was significantly reduced by reducing iron loading from 10 to 6% and 3%. They also stated that the amount of CO_2_ produced due to the degradation of acetaldehyde was subsequently reduced ^6^. In 2018, Yao et al. stated in their research for the photocatalytic removal of styrene, despite the wide band gap of TiO_2_ nanoparticles, its weak activity in the visible range has limited its application. They used TiO_2_-UiO-66-NH_2_ nanocomposite with weight percentages of 5%, 10% and 15% TiO_2_ to remove volatile organic compounds. Compared with TiO_2_, the TiO_2_-UiO-66-NH_2_ nanocomposite can increase the absorption of light to the visible range and accelerate the removal of generated electron holes. It was observed that TiO_2_-UiO-66-NH_2_ nanocomposite with 5% by weight of TiO_2_ can remove more than 99% of styrene after 600 min, while TiO_2_ alone can only remove 32.5% of it ^8^.

#### The effect of temperature

Another environmental parameter that can be effective in the degradation process is temperature. As shown in Fig. 7, the degradation rate increased with increasing temperature. The rate of degradation at 20 °C, 25 °C, and 35 °C was 53%, 61%, and 69%, respectively, which could be due to the fact that with increasing temperature, the amount of activity and collision of particles increases, and they can hit more active sites and the number of destruction increases. Jeong et al. in their study regarding acetaldehyde removal by FexOyHz/Al_2_O_3,_ observed that the decrease in temperature resulted in decreasing the amount of produced CO_2_ and the gas degradation efficiency ^6^. Although the detailed information about the effects of temperature used by our study has been reported elsewhere ^57^, we briefly have stated based on Eq. 2, according to the Arrhenius equation, increasing the temperature has a positive effect on the reaction kinetics ^58,59^.

2 $${\text{k}} = {\text{f}}\left( {{\text{exp}}\left( { - {\text{E}}/{\text{RT}}} \right)} \right)$$

In this study, K: Arrhenius temperature constant, E: energy, T: temperature and R: universal gas constant (8.314 × 10 kJ.mol^-1^ K^-1^).

In a study conducted to remove organic compounds by Cai et al. it was observed that their concentration decreased with increasing temperature ^60^.

#### The effect of relative humidity

Air humidity is one of the effective factors in the photocatalytic removal of pollutants; Because water molecules are the source of production of hydroxyl radicals, which decrease the amount of production of hydroxyl radicals on the surface of the catalyst and the efficiency of photocatalytic degradation. Based on Eqs. 3 and 4, water molecules on the surface of the catalyst are oxidized to hydroxyl radicals and lead to the enhancement of the photocatalyst reaction ^61^. In this study, the amount of humidity in different amounts was investigated. As predicted, with increasing humidity from 15 to 30% and 45%, the removal rate initially increased from 47 to 61% and then decreased to 55% (Fig. 7). According to other studies, this decrease in the degradation percentage is due to increased water vapor, the filling of nanoparticle holes, and their decrease in volume. Therefore, the rate of pollutant exposure to active sites is reduced, and the degradation percentage is reduced. Similarly, it was observed in another study that by increasing the humidity from 0 to 30%, the acetaldehyde removal efficiency decreased, and more acetaldehyde was observed at the reactor outlet ^6^. Moreover, Jiang et al. revealed that the degradation efficiency of volatile organic compounds increased with increasing humidity due to the production of hydroxyl radicals. However, the removal efficiency decreased with increasing humidity and extra water. This reduction in degradation efficiency is due to the electronegativity of water molecules, which limits the density of electrons and chemically active molecules ^61^. Also, in another study that was conducted to remove toluene with TiO_2_ nanoparticles by Sleiman et al., it was observed that the removal efficiency increased with the increase of humidity up to 30%, but then the removal efficiency decreased with the increase of humidity up to 80% ^62^. In another study, the researchers reported that increasing the humidity to some extent due to the production of hydroxyl radicals increases the degradation rate, but increasing it too much has a negative effect and reduces the removal efficiency ^54^. Therefore, it is necessary to maintain an optimal amount of water vapor in the system.

3 $${\text{MIL 1}}00\left( {{\text{Fe}}} \right)\left( {{\text{h}}_{{{\text{yb}}}}^{ + } } \right) + {\text{H}}_{{2}} {\text{O}} \to {\text{MIL 1}}00\left( {{\text{Fe}}} \right) + {\text{H}}^{ + } + {\text{OH}}^{ - }$$

4 $${\text{MIL 1}}00\left( {{\text{Fe}}} \right)\left( {{\text{h}}_{{{\text{yb}}}}^{ + } } \right) + {\text{OH}}^{ - } \to {\text{MIL 1}}00\left( {{\text{Fe}}} \right) + {\text{OH}}^{ \circ }$$

#### The effect of irradiation time

As indicated in Fig. 7, time is an important parameter in the process of photocatalytic degradation. This study examined the duration of 30 min, 60 min, 90 min, and 120 min. It was observed that increasing the time leads to increased degradation. The highest percentage of styrene degradation was obtained after 2 h at 89%. According to other studies, increasing the time allows more pollutants to come into contact with the catalyst, which leads to more pollutant degradation. Jiang et al. observed that the degradation efficiency of volatile organic compounds increased with increasing retention time. They stated that the degradation rate was extremely high; however decreased over time ^61^. Young Lee et al. also observed in their study titled photocatalytic removal of pollutants using TiO_2_-SiO_2_ nanocomposite that the removal efficiency increased with increasing retention time ^21^. As the retention time increases, the removal efficiency may increase; however, it should also be economically viable and not lead to a waste of energy and capital.

#### Photocatalytic degradation mechanism

**Figure 8:**
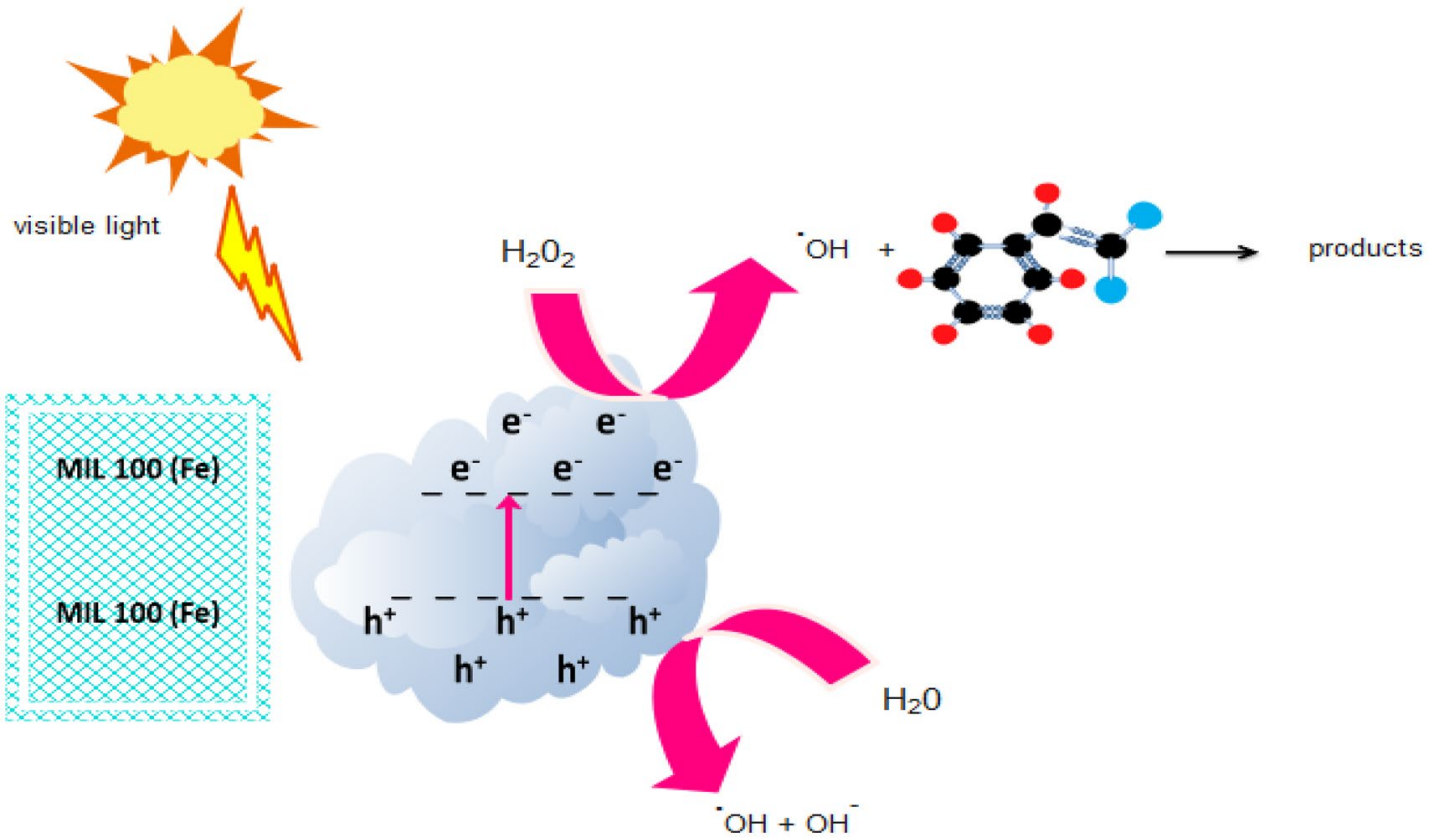
Photocatalytic degradation process of styrene by MIL100 (Fe) nanoparticles coated on glass under visible light.

In the photocatalytic mechanism, electrons (e^-^) are transferred from the capacitance band to the conduction band, and holes (h^+^) are created in the capacitance band of MIL100 (Fe). The holes created in the capacitance band react directly with OH^-^ or H_2_O to form hydrogen peroxide, facilitating the photocatalytic process. The H_2_O_2_ can react with MIL100 (Fe) as an electron receptor to produce ^·^OH radicals. Therefore, hole (h^+^) and ^·^OH radicals can lead to the decomposition of adsorbed organic compounds (Fig. 8 and Eqs. (5–8))^50^. In a study conducted by Zeng et al. in order to investigate the efficiency of Ag-TiO_2_ nanocomposite in the degradation of gaseous acetaldehyde under fluorescent light irradiation in 2018, they observed that the radical O_2_^·−^ had the main role in the degradation and decomposition of acetaldehyde^30^.

5 $${\text{MIL 1}}00\left( {{\text{Fe}}} \right) + {\text{h}}\nu \to {\text{h}}^{ + } \left( {{\text{MIL 1}}00\left( {{\text{Fe}}} \right)} \right)^{ \cdot } + {\text{e}}^{ - } \left( {{\text{MIL 1}}00\left( {{\text{Fe}}} \right)} \right)$$

6 $${\text{e}}^{ - } + {\text{O}}_{{2}} \left( {{\text{adsorbed}}} \right) \to {\text{O}}_{{2}}^{ \cdot - }$$

7 $${\text{h}}^{ + } + {\text{H}}_{{2}} {\text{O}} \to \cdot {\text{OH}} + {\text{H}}^{ + }$$

8 $${\text{O}}_{{2}}^{ \cdot - } + \, ({\text{h}}^{ + } / \cdot {\text{OH}}) + {\text{Styrene}} \to {\text{degradation products}}$$

**Figure 9:**
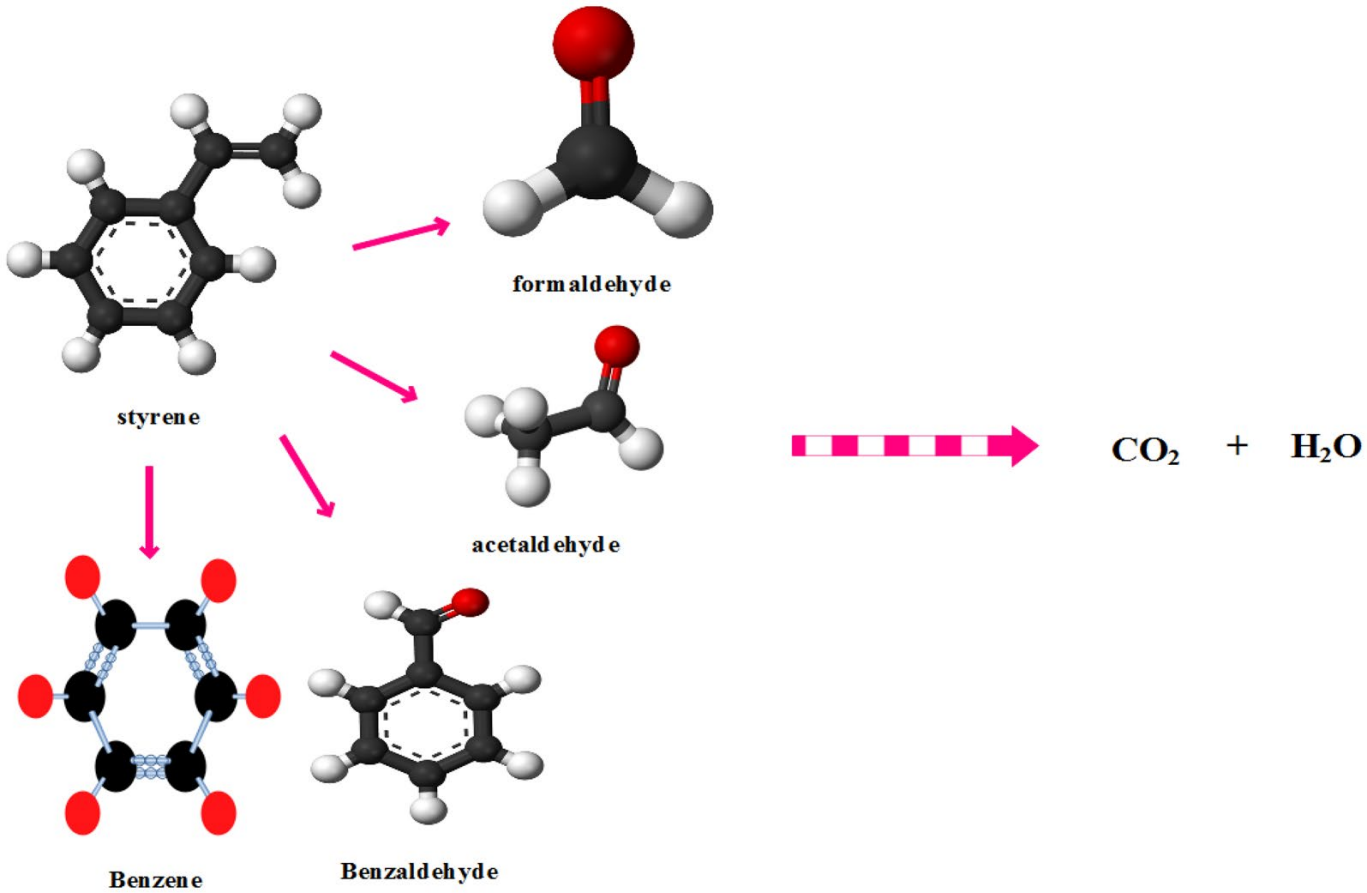
Byproducts of styrene degradation.

According to the above equations, two active radicals O_2_^·−^ and ^·^OH play a major role in the photocatalytic degradation of styrene. These radicals cause the breakage of styrene bonds and the production of by-products such as benzene, benzaldehyde, acetaldehyde, and formaldehyde, which finally decomposes into H_2_O and CO_2_ (Fig. 9). The generated radicals attack styrene. The bond energy of C–H, C–C and C=C is 4.3 eV, (5.0–5.3) eV and 5.5 eV respectively^24^. Decomposition of styrene takes place by dehydrogenation of ^·^OH. In this way, energetic molecules hit the reaction sites and lead to the activation of that site and decomposition of styrene. Catalysts MIL100 (Fe) due to their high specific surface area and fast transport, reduce the deposition of intermediate products and significant photocatalytic activity. Also, the photocatalytic process with light radiation and the production of active radicals causes the mineralization of manufactured products into H_2_O and CO_2_. Nevertheless, after some time due to the production of competitive elimination intermediate products, it causes a slight decrease in styrene degradation. At the beginning of the photocatalytic degradation process, the amount of carbon dioxide production was low, but as the irradiation time increased and the removal efficiency increased, the amount of carbon dioxide production increased, which indicates that the presence of the catalyst causes mineralization of the pollutant.

#### Kinetics of photocatalytic degradation of styrene

The photocatalytic degradation kinetics of MIL100 (Fe) were performed for analysis under optimal conditions (concentration of 0.6 g/l of MIL100 (Fe), the temperature of 35 °C, relative humidity of 30%, and pollutant concentration of 50 ppm). Following previous studies, first and second order kinetics were employed to examine the photocatalytic degradation of styrene. According to Eqs. (9,10), C_0_ is the initial styrene concentration, and C_t_ is the initial styrene concentration at time t. K_0_ denotes the first and second-order reaction rate constant (min^-1^), and t implies the photocatalytic reaction time. K (reaction rate constant) is obtained from the quasi-first-order rate curve.

9 $$\ln \frac{{C_{0} }}{{C_{t} }} = K_{0} {\text{t}}$$

10 $$k{\text{t}} = \frac{1}{{c_{t} }} - \frac{1}{{c_{0} }}$$

The results of photocatalytic degradation of MIL100 (Fe) are presented in Table (2). As can be seen, the coefficient value is approximately equal to 1, which indicates that first-order kinetics is the best model for expressing the amount of pollutant degradation because the degradation of styrene depends on the catalyst dose. According to the results, MIL100 (Fe) catalyst had the highest destruction rate (K_0_ = 1.51 × 10^–2^ min^-1^) due to the high area determined by BET analysis. This efficiency is due to the easy separation of the electron–hole pair resulting from the rapid transfer of MIL100(Fe) electrons.

Similar to the present study, Mahmoodi et al., in their study on the color degradation of textile factory wastewater using MIL100(Fe) organic–metallic framework under ultraviolet light irradiation, observed that the degradation kinetics followed the first-order equation and the degradation rate for concentrations of 0.01, 0.02, 0.03, and 0.04 g of the catalyst obtained 0.0034, 0.0041, 0.0091, and 0.0289 l/min, respectively ^45^. Wang et al. also reported that the elimination reaction rate obeyed the first-order kinetics in the research they conducted for the degradation of tetracycline using Fe-MILs (Table 2)^30^.

**Table 2:** Parameters on the kinetic of the styrene degradation process of MIL100(Fe).

MOF	Equation	k_0_ (min^−1^)	R^2^
MIL100(Fe)	y = 0.0151x + 0.352	1.51 × 10^–2^	0.9877
MIL100(Fe)	y = 0.0445x − 0.031	4.45 × 10^–2^	0.9337

Thermodynamic parameters such as entropy (∆S°), Gibbs free energy (∆G°) and enthalpy (∆H°) for styrene degradation with nanoparticles were calculated from the change of Kc with temperature. (Eqs. 11–14)^63^

11 $$\Delta {\text{G}}^\circ = \Delta {\text{H}}^\circ - {\text{T}}\Delta {\text{S}}^\circ$$

12 $$\Delta {\text{G}}^\circ = - {\text{RTLn Kc}}$$

13 $${\text{Kc}} = \frac{{{\varvec{Ceq}}}}{{{\varvec{CAe}}}}$$

14 $${\text{Ln Kc}} = \frac{{\Delta {\text{S}}^\circ }}{R} - \frac{{\Delta {\text{H}}^\circ }}{RT}$$

**Figure 10:**
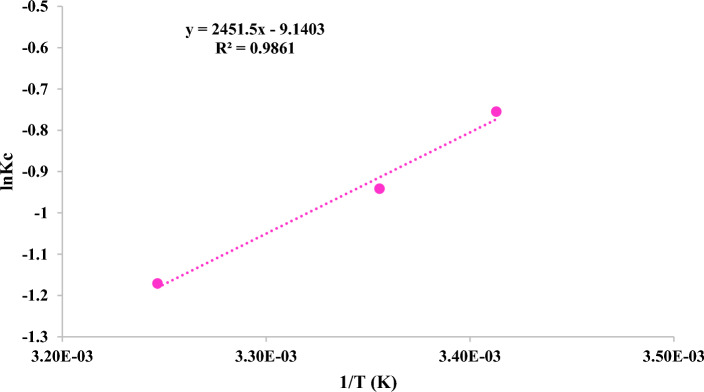
Experimenting the thermodynamic parameters of styrene degradation.

∆S° entropy change (kJ mol^−1^), ∆G° free energy change (kJ mol^−1^) and ∆H^o^ enthalpy change (kJ mol^−1^), Ceq = pollutant concentration in equilibrium, Ae = pollutant concentration removed is in equilibrium. Thermodynamic parameters are given in Table 3. The negative value of ∆H^o^ indicates that the process of styrene degradation by MIL100(Fe) nanoparticles is exothermic. ∆S° was obtained for the concentration of 50 ppm pollutant − 0.0759925 (kJ mol^−1^). With the increase in temperature due to the molecular motion, the amount of ∆G° increased from 1.839237 (kJ mol^−1^) at 293°k, to 2.332903 (kJ mol^−1^) at 298°k and 2.999062 (kJ mol^−1^) at 308°k temperature (Fig. 10). The findings indicate that the value of ∆G° increased with increasing temperature.

**Table 3:** Termodynamic parameters in the styrene degradation process by MIL100(Fe) for different temperatures.

Styrene concentration (ppm)	∆H^o^ (kJ mol^−1^)	∆S° (kJ mol^−1^)	∆G° (kJ mol^−1^)		
			293 k	298 k	308 k
50	− 20.3818	− 0.0759925	1.839237	2.332903	2.999062

#### Cost and real-time analysis

In this study, For the synthesis of 3 g of nanoparticles, Fe(NO_3_)_3_·9H_2_O, C_9_H_6_O_6_, methanol was purchased for $27. In order to analyze nanoparticles to determine their characteristics, $20 was paid. Also, to prepare a pseudo-reactor; The glass, fan, lamp, aluminium foil,… was purchased for $35. In order to determine the amount of pollutant degradation under optimal conditions, first the test was conducted under conditions of temperature, humidity, pollutant concentration, nanoparticle concentration and time of 120 min. In order to prepare the reactor, first the air was removed for 10 min by the log pump. After checking the flow rate of 1 L, the inlet flow entered Erlen. Again, the flow rate was checked by rotameter before entering the reactor. The amount of input pollutant was checked so that the desired amount entered the reactor. After reaching that level of concentration, the control valve is closed and the destruction process is carried out in the determined time, and after that period, the output amount of the pollutant is determined. All these steps lasted for 3 h.

## Conclusion

Volatile organic compound styrene, which is mainly used in plastic production industries, is classified as a possible carcinogen according to the reports of various organizations. Its removal from polluted air has been considered. Different methods have many advantages and disadvantages. The process of photocatalytic degradation with nanoparticles MIL100(Fe) a new and biocompatible method due to the supply of energy by the sun and cheap due to the use of iron as an abundant element in the environment, which had a high efficiency under the conditions of temperature and humidity of the environment. This nanoparticle has been used in water environments and it has rarely been used to destroy air pollutants. The photocatalytic efficiency of MIL100(Fe) coated on the glass to remove styrene was investigated. The results indicates that this process provides excellent efficiency for the photocatalytic removal of styrene. The increase in degradation by adding nano-catalysts is due to the porous structure of MIL100(Fe), which accelerates the electron transfer conditions. The degradation kinetics followed the first-order kinetic model. According to the results obtained from BET analysis, it was observed that the MIL100(Fe) catalyst had a large pore size and volume. It was observed that the removal efficiency of styrene was inversely related to the number of input pollutants. As the concentration increased from 50 to 150 ppm, the removal efficiency decreased from 61 to 39%. Considering that as the duration of light irradiation to the surface of the photocatalyst increased, the removal efficiency increased from 58% in 30 min to 89% in 120 min; It can be said that the photocatalytic process is highly efficient in conditions where the duration of light irradiation to the surface of the photocatalyst is longer. The highest removal rate of styrene was 89% at 50 ppm, 30% humidity, a temperature of 35 °C, and a nanoparticle dosage of 0.6 g/l after 120 min of light irradiation. It can be concluded that MIL100 (Fe), as a nature-friendly photocatalyst with unique properties, can be coated on pavements, floors, building materials, etc. to remove other pollutants in Gas phase is used.

## Data Availability

All data generated or analyzed during this study are included in this published article.
